# Sympathoexcitation and impaired arterial baroreflex sensitivity are linked to vascular inflammation in individuals with elevated resting blood pressure

**DOI:** 10.14814/phy2.14057

**Published:** 2019-04-09

**Authors:** Ida T. Fonkoue, Ngoc‐Anh Le, Melanie L. Kankam, Dana DaCosta, Toure N. Jones, Paul J. Marvar, Jeanie Park

**Affiliations:** ^1^ Renal Division Department of Medicine Emory University School of Medicine Atlanta Georgia; ^2^ Research Service Line Atlanta VA Healthcare System Decatur Georgia; ^3^ Biomarker Core Laboratory Atlanta VA Healthcare System Decatur Georgia; ^4^ Department of Pharmacology and Physiology Institute for Neuroscience George Washington University Washington District of Columbia

**Keywords:** Baroreflex, blood pressure, inflammation, MSNA

## Abstract

Elevated Resting Blood Pressure (ERBP) in the prehypertensive range is associated with increased risk of hypertension and cardiovascular disease, the mechanisms of which remain unclear. Prior studies have suggested that ERBP may be associated with overactivation and dysregulation of the sympathetic nervous system (SNS). We hypothesized that compared to normotensives (≤120/80 mmHg), ERBP (120/80–139/89 mmHg) has higher SNS activity, impaired arterial baroreflex sensitivity (BRS), and increased vascular inflammation. Twenty‐nine participants were studied: 16 otherwise healthy individuals with ERBP (blood pressure (BP) 130 ± 2/85 ± 2 mmHg) and 13 matched normotensive controls (mean BP 114 ± 2/73 ± 2 mmHg). We measured muscle sympathetic nerve activity (MSNA), beat‐to‐beat BP, and continuous electrocardiogram at rest and during arterial BRS testing via the *modified Oxford* technique. Blood was analyzed for the following biomarkers of vascular inflammation: lipoprotein‐associated phospholipase A2 (Lp‐PLA2), E‐selectin, and intercellular adhesion molecule 1 (ICAM‐1). Resting MSNA burst frequency (22 ± 2 vs. 16 ± 2 bursts/min, *P* = 0.036) and burst incidence (36 ± 3 vs. 25 ± 3 bursts/100 heart beats, *P* = 0.025) were higher in ERBP compared to controls. Cardiovagal BRS was blunted in ERBP compared to controls (13 ± 2 vs. 20 ± 3 msec/mmHg, *P* = 0.032), while there was no difference in sympathetic BRS between groups. Lp‐PLA2 (169 ± 8 vs. 142 ± 9 nmol/min/mL,* P* = 0.020) and E‐selectin (6.89 ± 0.6 vs. 4.45 ± 0.51 ng/mL,* P* = 0.004) were higher in ERBP versus controls. E‐selectin (*r* = 0.501, *P* = 0.011) and ICAM‐1 (*r* = 0.481, *P* = 0.015) were positively correlated with MSNA, while E‐selectin was negatively correlated with cardiovagal BRS (*r* = −0.427, *P* = 0.030). These findings demonstrate that individuals with ERBP have SNS overactivity and impaired arterial BRS that are linked to biomarkers of vascular inflammation.

## Introduction

Chronic elevations in resting blood pressure (BP) in the prehypertensive range (BP of 120–139/80–89 mmHg) have been consistently reported as an independent risk factor for the development of hypertension and other cardiovascular diseases (CVD) such as atherosclerosis (Liszka et al. 2005; Atilla and Vasan 2006). While this range of elevated resting blood pressure (ERBP) is defined by the Joint National Commission (JNC) 7 as prehypertension (Chobanian et al. 2003), and as elevated BP (120–129/80–89) and Stage I hypertension (130–139/80–89) as per the American College of Cardiology/American Heart Association (ACC/AHA) guidelines (Whelton et al. 2018), the current recommendation and clinical approach for otherwise healthy patients that fall in this range of ERBP, whether defined as prehypertension or Stage I hypertension, is to institute lifestyle modifications without starting antihypertensive drug therapy (Chobanian et al. 2003; Whelton et al. 2018). The pathophysiological mechanisms by which ERBP, even in the absence of other CVD risk factors, is independently related to increased CVD risk are not clearly established, and identifying such mechanisms could inform therapeutic targets of early intervention in this at‐risk patient population, before the development of overt hypertension (Collier and Landram 2012).

Although the mechanisms underlying increased CVD risk in prehypertension remain unclear, prior studies, although controversial, suggest that ERBP may be associated with autonomic alterations (Davis et al. 2012) including chronic activation of the sympathetic nervous system (SNS). Some (Matsukawa et al. 1991; Davis et al. 2012), although not all (Schwartz et al. 2011) existing reports have shown that prehypertension is associated with SNS overactivity and decreased vagal modulation. One potential mechanism that could contribute to SNS overactivation and progression to overt hypertension in ERBP is impaired arterial baroreflex sensitivity (BRS) (Bristow et al. 1969), the major regulatory system controlling SNS output. Blunted arterial BRS has been described in other patient populations characterized by increased CVD risk including hypertension, heart failure, atherosclerosis, and chronic kidney disease, and is independently associated with poorer clinical outcomes (Bristow et al. 1969; Johansson et al. 2005; Nasr et al. 2005). However, whether arterial BRS is impaired in ERBP is unclear. Experimental studies (Gordon and Mark 1984) have reported impaired baroreceptor discharge in prehypertensive Dahl salt‐sensitive rats compared to normotensive rats (Gordon and Mark 1984), while human studies have shown mixed results (Kotchen et al. 1989; Rea and Hamdan 1990; Pal et al. 2015), likely due in part to differences in technique and study populations including racial differences. A major goal of this study was to determine if ERBP is characterized by overactivation of the SNS and impaired arterial BRS using direct intraneural measures of SNS activity and pharmacologic manipulation of arterial BP in a predominantly African‐American (AA) study population.

In addition, one potential mechanism that could contribute to both SNS overactivity and impaired BRS is chronic inflammation, particularly vascular inflammation. Prior studies have suggested that inflammation (Takagishi et al. 2010; Marvar et al. 2016; Fonkoue et al. 2018) and endothelial remodeling (Savoia et al. 2011) may have a role in impairing arterial BRS at the level of the nucleus tractus solitarii in the brainstem, or the baroreceptor nerve endings within the vasculature, thereby contributing to the maintenance of SNS overactivation and subsequent development of hypertension (Bristow et al. 1969; Marvar et al. 2016). Lipoprotein‐associated phospholipase A2 (Lp‐PLA2) is a biomarker for atherogenesis that is highly specific for vascular inflammation and has low biological variability (Madjid et al. 2010). In addition, cell adhesion molecules (CAMs), expressed by activated endothelial cells, including E‐selectin (adhesion molecule expressed by endothelial cells) and intercellular adhesion molecule 1 (ICAM‐1) are biomarkers of vascular inflammation that are associated with CVD outcomes (Krieglstein and Granger 2001; Eikendal et al. 2018). While studies have suggested that prehypertension may be associated with increased systemic inflammatory markers such as C‐reactive protein and interleukin‐6 (Nandeesha et al. 2015), we tested the hypothesis that individuals with ERBP have higher levels of biomarkers specific for vascular inflammation, and that these biomarkers would be linked to sympathetic overactivation and impaired arterial BRS in ERBP. Characterizing sympathetic activity, BRS and vascular inflammation in ERBP could add new information about potential mechanisms linking prehypertension and CVD risk.

## Methods

### Ethical approval

This study conformed to the standards set by the Declaration of Helsinki and was approved by the Emory University Institutional Review Board and the Atlanta Veterans Affairs Health Care System Research and Development Committee. All participants provided written informed consent for study participation via forms approved by the aforementioned regulatory committees.

### Study population

The study population consisted of 29, predominantly African‐American participants: 16 with ERBP and 13 matched normotensive controls. Normotension was defined as a resting BP of <120/80 mmHg as per the JNC 7 and ACC/AHA guidelines, and ERBP was defined as a resting BP of 120–139/80–89 mmHg, also termed prehypertension per JNC 7 (Chobanian et al. 2003) and elevated BP + Stage I hypertension per ACC/AHA guidelines (Whelton et al. 2018). All study participants were otherwise healthy without pharmacologically treated hypertension or other medical comorbidities. All participants were ambulatory, recreationally active but not trained athletes, and free of systemic diseases. Exclusion criteria for all participants included the use of antihypertensive medications, smoking, diabetes, heart or vascular disease, illicit drug use, excessive alcohol use (>1–2 drinks per day), hyperlipidemia, autonomic dysfunction, medications known to affect SNS (clonidine, *β*‐blockers, angiotensin‐converting enzyme inhibitors, stimulants, and antidepressants such as selective serotonin and norepinephrine reuptake inhibitors), treatment with monoamine oxidase inhibitors, posttraumatic stress disorder, and any systemic disease.

### Measurements and procedures

#### Blood pressure (BP), heart rate (HR), and respiratory rate (RR)

Three seated BP measurements separated by 5 min were taken during at least 2–3 separate screening visits to confirm normotension versus ERBP status. The screening visits blood pressure was taken after 10 min of quiet, seated rest. A single study coordinator took all BP measurements using the standard ACC/AHA guideline technique (Whelton et al. 2018) with an appropriately sized cuff placed on the upper arm with the arm resting at heart level using an automated digital BP device (Omron, HEM‐907XL, Omron Healthcare, Kyoto, Japan). During the experimental protocol, beat‐to‐beat arterial BP was measured continuously and noninvasively using digital pulse photoplethysmography (CNAP Monitor 500, CNSystems, Graz, Austria) as previously described (Fonkoue et al. 2018). Absolute values of BP were calibrated with upper arm BP readings using an internal calibration system at the start and every 15 min throughout the study. This device has been validated to reflect accurate absolute and beat‐to‐beat fluctuations in BP when compared with invasive intra‐arterial catheter readings (Ilies et al. 2012). HR was measured using continuous electrocardiography with a Bio Amp (model ML 132, ADInstruments, Colorado Springs, CO). Respiratory rate (RR) was continuously monitored via a respiratory belt pressure transducer placed around the upper abdomen (Pneumotrace II, ADInstruments).

#### Muscle sympathetic nerve activity (MSNA)

Multiunit postganglionic sympathetic nerve activity directed to muscle (MSNA) was recorded using microneurography, as described previously (Wallin and Fagius 1988). A tungsten microelectrode (tip diameter 5–15 *μ*m; Bioengineering, University of Iowa, Iowa City, IA) was inserted into the peroneal nerve with a reference microelectrode inserted in close proximity. The efferent nerve signals were amplified (total gain 50,000–100,000), filtered (700–2000 Hz), rectified, and integrated (time constant 0.1 sec) to obtain a mean voltage display (Nerve Traffic Analyzer, model 662C‐4; Bioengineering, University of Iowa) of MSNA that was recorded using LabChart 7 (PowerLab 16sp, ADInstruments, Sydney, Australia) along with continuous ECG, BP, and RR. All MSNA recordings met previously established quality standards (Mano et al. 2006). MSNA was ascertained by verifying entrainment to the HR with bursts falling between R‐R intervals with a 3:1 burst‐to‐baseline ratio and activity increasing with apnea and Valsalva maneuver. Skin sympathetic nerve activity was also ruled out by verifying the lack of reactivity to acoustic startle. All MSNA data were analyzed by a single investigator blinded to the participant's group status as ERBP or normotensive.

#### Arterial baroreflex sensitivity (BRS) using the Modified Oxford technique

The gold‐standard method for evaluation of arterial BRS was performed by measuring changes in MSNA and R‐R interval during arterial BP changes induced by sodium nitroprusside (NTP) and phenylephrine (PE) (Rudas et al. 1999). NTP 100 *μ*g in 10 mL of normal saline (NS) was bolused through an antecubital intravenous (IV) catheter, followed 60 sec later by an IV bolus of PE (150 *μ*g in 10 mL of NS) during continuous MSNA, ECG, and hemodynamic monitoring. Medications were at room temperature at the time of administration. These medications induce a decrease of ~15 mmHg (NTP effect), followed by an increase above baseline of ~15 mmHg (PE effect) in arterial BP (Bonyhay and Freeman 2004).

#### Vascular inflammatory biomarkers

Venous blood samples were collected in all participants on the day of the experiment prior to any instrumentation, for the following inflammatory biomarkers: lipoprotein‐associated phospholipase A2 (Lp‐PLA2), E‐selectin, and ICAM‐1. Blood was collected in EDTA‐treated whole blood tubes and was centrifuged for 15 min at 2200–2500 RPM. The collected plasma samples were stored at −80°C for subsequent immunohistochemical analyses.

#### Lp‐PLA2 activity

The activity of Lp‐PLA2, also known as platelet‐activating factor acetylhydrolase (PAF‐AH) was measured at the Biomarker Core Laboratory of the Atlanta VA, using a PAF‐AH activity assay kit, which produces results based on a colorimetric shift (Biovision, Milpitas, CA), according to the manufacturer's instructions. Performance characteristics for the Lp‐PLA2 test were established using the enzymatic method Beckman Coulter AU400, a dual monoclonal antibody immunoassay standardized to recombinant Lp‐PLA2 (PLAC test, diaDexus, Inc) (Dada et al. 2002). To assess intra‐assay precision and total variability for Lp‐PLA2 measurement, five human plasma samples and two buffer controls with Lp‐PLA2 activity distributed throughout the calibration range of the assay were measured in 40 separate assays to determine the intra‐assay coefficient of variation (Le et al. 2015).

#### E‐selectin and ICAM‐1

Adhesion molecules ICAM‐1 (cluster of differentiation (CD)54; intercellular adhesion molecule‐1), and E‐selectin (CD62E; E‐SEL) were measured in duplicate in the biomarker core laboratory at Emory University, using the Meso Scale Discovery (MSD) human multiplex ELISA. This electrochemiluminescence system has been validated by comparisons with traditional ELISA and produces measurements that have high content validity (Fichorova et al. 2008; Leviton et al. 2011). Human E‐selectin and ICAM‐1 in plasma were measured using the human multiple V‐PLEX kit (Meso Scale Discovery, Rockville, MD) according to the manufacturer's protocol. The signals were read on the MSD QuickPlex instrument and the concentration was evaluated on the MSD software platform.

### Experimental protocol

On the study date, participants presented after abstaining from food and caffeine for 12 h, and alcohol, medications, and exercise for at least 24 h. After obtaining a seated BP measurement, an IV catheter was placed in the antecubital vein for blood draws and for subsequent boluses of NTP and PE during BRS testing. Participants were placed in a supine position on a comfortable stretcher and fitted with ECG electrodes, finger cuffs for continuous hemodynamic monitoring, and a respiratory belt sensor to measure continuous RR. Microneurography was performed as described above. After a satisfactory MSNA neurogram was obtained, the participant rested for 15 min. Baseline MSNA, ECG, and beat‐to‐beat BP were then measured and recorded for 10 min, followed by pharmacological manipulation of BP for BRS assessment using the modified Oxford technique as described above.

### Data analysis

#### Hemodynamics, muscle sympathetic nerve activity, and BRS assessment

For all baseline analyses, we used the last 5 min of the 10‐min baseline recording to ensure true resting state. MSNA, BP, and ECG data were exported from Labchart to WinCPRS (Absolute Aliens, Turku, Finland) for analysis as previously described (Park et al. 2017). The continuous arterial BP waveforms were analyzed for beat‐to‐beat changes in systolic BP (SAP), diastolic BP (DAP), and mean arterial pressure (MAP). R‐waves were detected and marked from the continuous ECG recording. MSNA bursts were automatically detected by the program using the following criteria: burst‐to‐noise ratio of 3:1 within a 0.5‐sec search window with an average latency of 1.2–1.3 s in burst occurrence from the previous R‐wave. After automatic detection, the ECG and MSNA neurograms were visually inspected for accuracy of detection by a single investigator without knowledge of the ERBP versus normotension status. MSNA was expressed as burst frequency (bursts per minute) and burst incidence (bursts per 100 heartbeats). Sympathetic BRS was quantified as the slope of the linear relationship between MSNA burst incidence and DAP during pharmacological manipulation of BP. Cardiovagal BRS was quantified as the slope of the linear relationship between R‐R interval and SAP. Only slope values with a correlation value >0.5 were included. At least 2 min of data (Park et al. 2017), including both the decreases and increases in BP induced pharmacologically were analyzed for each overall BRS assessment.

#### Statistical analysis

Data were analyzed using SPSS 22.0 (IBM SPSS, Armonk, NY). A chi‐squared test for independence was used to compare the categorical variables of race, sex, and family history of hypertension. An independent *t*‐test was performed to compare the continuous variables, including resting hemodynamics, Lp‐PLA2, E‐selectin, ICAM‐1, MSNA, and BRS between ERBP and controls. Pearson's correlation coefficients were calculated to examine the relationships between variables. Significance level was set at *α* < 0.05.

## Results

### Baseline characteristics

Twenty‐nine participants (16 with ERBP and 13 normotensive controls) were enrolled in the study. Resting SAP ranged from 120 to 138 mmHg for the ERBP group and 108 to 119 mmHg for the control group; DAP ranged from 77 to 90 mmHg for the ERBP group and 62 to 78 mmHg for the control group. Groups were well matched for age with a mean  ±  SE age of 36  ±  2 years in participants with ERBP and 33  ±  2 years in normotensive controls (Table 1). There were also no significant differences in sex, race, family history of hypertension, and body mass index (BMI) between groups. The majority of participants in each group were African‐American. Resting SAP and DAP were significantly higher in the ERBP group compared to controls as expected (*P* < 0.001), but heart rate (*P* = 0.382) and respiratory rate (*P* = 0.413) were comparable between the groups.

**Table 1:** Baseline characteristics of study population

Variable	ERBP	Controls	*P*‐value
	*n *= 16	*n *= 13	
Age (years)	36 ± 2	33 ± 3	0.293
Sex (Male/Female)	13/3	8/5	0.961
Race (Black/White)	12/4	11/2	0.223
BMI (kg/m^2^)	28 ± 1	28 ± 2	0.827
FHH (Yes/No)	6/10	3/10	0.419
Baseline Hemodynamics			
SAP (mmHg)	130 ± 2	114 ± 2	<0.001
DAP (mmHg)	85 ± 2	73 ± 2	<0.001
HR (beats/min)	63 ± 3	63 ± 3	0.382
RR (breaths/min)	16 ± 1	15 ± 1	0.413

Values are mean ± SE; BMI, body mass index; FHH, family history of hypertension; SAP, systolic arterial pressure; DAP, diastolic arterial pressure; MAP, mean arterial pressure; HR, heart rate; RR, respiratory rate. All *P*‐values are two‐tailed.

### Baseline MSNA

**Figure 1:**
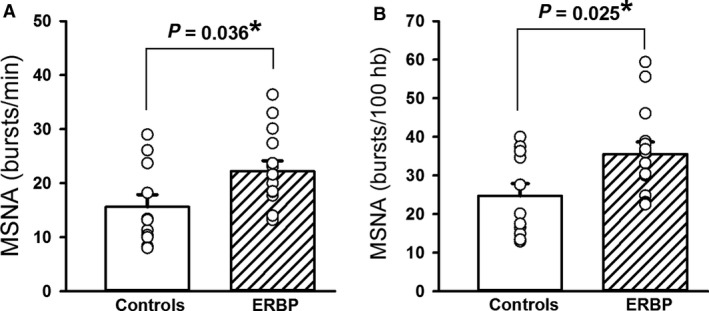
Muscle sympathetic nerve activity (MSNA) burst frequency (A) and burst incidence (B) at rest in 14 elevated resting blood pressure (ERBP) participants and 12 normotensive controls. Baseline MSNA frequency and MSNA incidence were higher in ERBP compared to controls. *P < 0.05.

As depicted in Figure 1, baseline MSNA was higher in the ERBP group compared to controls, quantified as burst frequency (*P* = 0.036) in A or burst incidence (0.025) in B. Suitable MSNA recordings were not obtained in two participants with ERBP and one normotensive control. Therefore, the results for MSNA are given for 14 ERBP and 12 controls participants.

### Baseline arterial BRS

**Figure 2:**
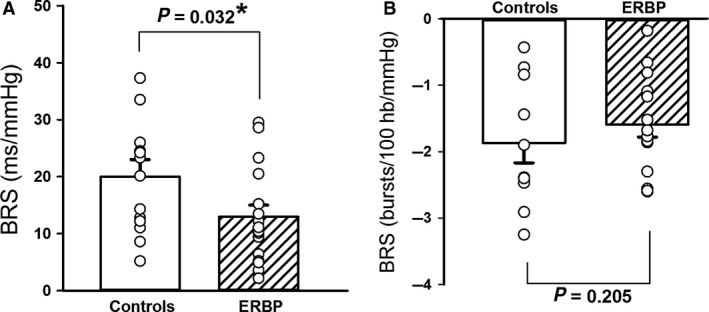
Cardiovagal and sympathetic baroreflex sensitivity (BRS) at rest. Mean baseline cardiovagal BRS (A) was lower in 16 elevated resting blood pressure (ERBP) participants compared to 13 normotensive controls. Baseline sympathetic BRS (B) was comparable between 14 ERBP and 12 controls. *P < 0.05.

Figure 2 shows the results for cardiovagal BRS (A) and sympathetic (B), quantified during pharmacological manipulation of BP as described above. At baseline, cardiovagal BRS assessed as the mean slope of the individual linear regressions between R‐R intervals and SAP was lower (*P* = 0.032) in ERBP compared to controls. However, there was no difference (*P* = 0.205) between the groups in sympathetic BRS assessed as the mean slope of the individual linear regressions between MSNA burst incidence and DAP. Separate analyses of BRS during the increasing BP phase versus the decreasing BP phase yielded comparable results to the overall BRS assessments in both ERBP and controls.

### Vascular inflammatory biomarkers

**Figure 3:**
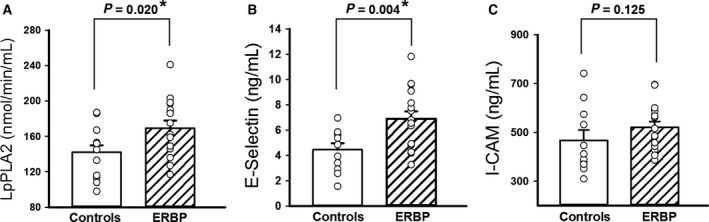
Lipoprotein‐associated phospholipase A2 (Lp‐PLA2) activity (A), E‐selectin (B) and intercellular adhesion molecule 1 (ICAM‐1) levels (C) in 14 elevated resting blood pressure (ERBP) participants and 12 normotensive controls. Mean baseline Lp‐PLA2 and E‐selectin were higher in ERBP compared to controls. ICAM‐1 tended to be higher in ERBP compared to controls. *P < 0.05.

**Figure 4:**
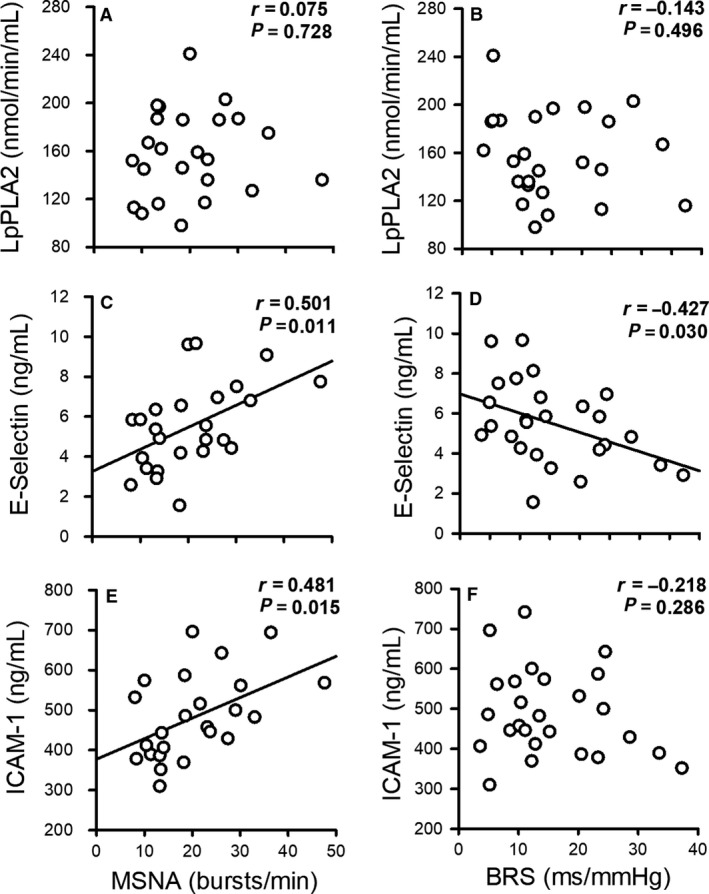
Correlation of resting muscle sympathetic nerve activity (MSNA) and cardiovagal baroreflex sensitivity (BRS) with lipoprotein‐associated phospholipase A2 (Lp‐PLA2) activity (A and B), E‐selectin (C and D), and intercellular adhesion molecule 1 (ICAM‐1) (E and F) in all participants. E‐selectin levels were positively correlated with MSNA and negatively correlated with cardiovagal BRS. ICAM‐1 levels were positively correlated with MSNA. The graphs with significant correlations (*P* < 0.05) are depicted with a regression line.

Figure 3 depicts the results of the vascular inflammatory biomarkers. Lp‐PLA2 (*P* = 0.020) was higher in ERBP compared to controls (A). E‐selectin (*P* = 0.004) was also higher in ERBP compared to controls (B), while ICAM‐1 tended to be higher in ERBP (C), although the difference between the groups did not reach significance (*P* = 0.125). Figure 4 shows the results of the correlations between inflammatory biomarkers, MSNA, and cardiovagal BRS. Lp‐PLA2 activity did not show a significant correlation with MSNA (*r* = 0.075, *P* = 0.728; A) or cardiovagal BRS (*r* = −1.43, *P* = 0.496; B). E‐selectin was positively correlated with MSNA (*r* = 0.501, *P* = 0.011; C) and negatively correlated with cardiovagal BRS (*r* = −0.427, *P* = 0.030; D). ICAM‐1 was also positively correlated with MSNA (*r* = 0.481, *P* = 0.015; E) but did not show a significant correlation with cardiovagal BRS (*r* = −0.218, *P* = 0.286; F). Results for inflammatory biomarkers are reported for 15 ERBP and 12 controls participants because blood samples were not obtained in two participants.

## Discussion

The mechanisms underlying increased CVD risk in patients with ERBP in the prehypertensive range remain to be fully understood. This study explored the potential roles of sympathetic overactivity, blunted baroreflex sensitivity, and vascular inflammation in an otherwise healthy, predominantly African‐American cohort with ERBP. We report the following new findings: (1) patients with ERBP had higher MSNA at rest compared to normotensive controls; (2) cardiovagal BRS was impaired in ERBP; (3) Lp‐PLA2 and E‐Selectin levels were higher in ERBP than in controls; and (4) E‐selectin and ICAM‐1 levels were positively related to MSNA; and (5) E‐selectin was negatively correlated with cardiovagal BRS. Together, these changes suggest that ERBP is characterized by a state of heightened SNS activity and impaired arterial BRS that are linked to vascular inflammation; these autonomic and inflammatory derangements are present before the onset of overt hypertension.

Sympathetic overactivity, independent of arterial BP, is linked to poor clinical outcomes in hypertension and other cardiovascular diseases (Grassi et al. 2004, 2018; Charkoudian and Rabbitts 2009; Grassi 2010). However, whether prehypertension is characterized by chronic SNS overactivation has been controversial. In the present study, we showed that ERBP had higher resting MSNA compared to normotensive controls. Matsukawa et al. (1991) reported higher MSNA and plasma norepinephrine in nine borderline hypertensives compared to 11 normotensives. Similar findings were reported in mild‐to‐moderate essential hypertension (Grassi et al. 2018). Seravalle et al. (2015) reported that MSNA was significantly greater in high‐normal BP (BP 130–139/85–89 mmHg) than in normal BP (120–129/80–84 mmHg) and optimal BP (BP < 120/80 mmHg). This is comparable to our results showing that baseline MSNA was higher in the ERBP (120–139/80–89 mmHg) compared to controls (BP < 120/80 mmHg). It is possible that the high‐normal BP included in this category was driving the higher resting MSNA. In contrast, Schwartz et al. (2011) found no difference in resting MSNA in 17 prehypertensives compared to 18 matched normotensives. The inconsistency among these studies could be due to differing ranges of resting BP between the studies. Matsukawa et al. (1991) defined borderline hypertension as a seated SAP > 140 mmHg and/or DAP > 90 mmHg, and normotension as SAP < 130 mmHg and DAP < 80 mmHg. Schwartz et al. (2011) on the other hand defined prehypertension as systolic pressure of 120–139 mmHg and/or a diastolic pressure of 80–89 mmHg and normotension as BP < 120/80 mmHg, similar to the current study. However, although the current study used the same BP parameters as Schwartz et al., we found significantly higher resting MSNA in ERBP compared to controls. This could be due to differences in the study populations, such as age, sex, and race. The prehypertensive group in the previous report was younger (mean age 23 years) than the ERBP cohort included in the current study (mean age 36 years), and MSNA has been reported to increase with age (Jones et al. 1997), and to be positively correlated with elevations in diastolic BP in older men (Hart et al. 2009). In addition, a strength of the current study is the inclusion of primarily African‐American men, an understudied population at higher CVD risk, while prior studies have included primarily White men. Thus, there may be racial differences in SNS overactivity and dysregulation that may begin earlier during prehypertension in African‐Americans and contribute to progression to overt hypertension.

One mechanism that could contribute to chronic overactivation of the SNS is impaired arterial BRS. We observed in the present study that participants with ERBP had lower cardiovagal BRS compared to controls, but comparable sympathetic BRS. These results are similar to the findings of Seravalle et al. (2015) in which patients with Stage I hypertension (BP 130–139/80–89 mm Hg) had lower cardiovagal BRS that was associated with metabolic syndrome. The current results suggest that impaired cardiovagal BRS is present with even lower increases in resting BP and is associated with vascular inflammation. Our results, however, contrast with the findings of Kotchen et al. (1989) who reported no differences in cardiovagal BRS in a cohort of 13 Caucasian males with prehypertension compared to 12 matched normotensive controls. This difference might be due in part to the fact that Kotchen et al. used the original Oxford Technique with boluses of nitroprusside (NTP) and angiotensin II (Smyth et al. 1969), while the current study employed the modified Oxford technique with sequential boluses of NTP and phenylephrine (PE). This distinction is important to note because PE has little or no effect on carotid sinus wall contraction, but angiotensin does have an effect which might lead to differences in vascular responses (Boulanger et al. 1995). Furthermore, although it is unknown if there are racial differences in BRS, our cohort, was predominantly composed of Black men while the earlier study was conducted in White men. Impaired arterial BRS has been shown to contribute to the onset of hypertension (Del Colle et al. 2007; Sharman et al. 2018) and is independently linked to heightened CVD risk (La Rovere et al. 1998, 2001). Early studies on animal models showed that in Dahl salt‐sensitive rats, impaired BRS precedes any elevation in arterial BP and may contribute to the propensity of these rats to develop hypertension (Gordon and Mark 1984). Similar to the findings in animals, Pal et al. (2015) reported a decrease in cardiovagal BRS in salt‐preferring individuals, another population at risk for the development of hypertension. Thus, impaired arterial BRS may contribute to SNS overactivity, as well as increased risk for progression to hypertension and CVD in ERBP.

While we showed that cardiovagal BRS is blunted, we did not find a difference in sympathetic BRS between ERBP and normotensive controls. Discordant sensitivities between the two efferent arms of the baroreflex (cardiovagal and sympathetic) have been described in other populations including rheumatoid arthritis (Adlan et al. 2017), young males (Dutoit et al. 2010; Taylor et al. 2015), elderly individuals (Ebert et al. 1992; Okada et al. 2012), and hypertensives (Grassi et al. 1998; Seravalle et al. 2015). This dissociation might be explained by the fact that, although cardiac and sympathetic baroreflex responses share the same afferent input, there are unknown factors influencing the central integration of the afferent input and the efferent pathways (Taylor et al. 2015). It is possible that in ERBP, the impairment in the cardiovagal efferent precedes that of the sympathetic efferent branch, and that derangement in one of the two branches is sufficient to result in autonomic dysregulation.

We further explored whether vascular inflammation was increased in ERBP and could be an underlying mechanism for impaired BRS and SNS activation. Chronic inflammation could lead to BRS dysfunction that is compromised sensitivity at vascular nerve endings or along its afferent and efferent tracts in the central nervous system, prior to the development of overt CV disease (Chapleau et al. 2001). While studies have shown that some inflammatory biomarkers such as C‐reactive protein (CRP) and interleukin 6 (IL‐6) may be elevated in prehypertension, whether biomarkers specific for vascular inflammation are elevated in prehypertension are less clear. In the current study, we show that Lp‐PLA2 and E‐selectin levels, biomarkers of vascular inflammation, were significantly elevated in ERBP compared to controls. Lp‐PLA2, a member of the phospholipase A2 family of enzymes produced by circulating macrophages, T lymphocytes, and mast cells (Yang et al. 2006) is associated with vascular abnormalities including endothelial dysfunction and arterial stiffness (Ikonomidis et al. 2014; Kim et al. 2017), and shown in longitudinal studies to be a reliable predictor of cardiovascular events (Madjid et al. 2010). In a recent longitudinal study, Kim et al. (2017) observed significant increases in circulating Lp‐PLA2 activity after 3.5 years in individuals with persistent prehypertension compared to individuals whose BP status reverted to normotension, suggesting that vascular inflammation is associated with the maintenance of elevated resting BP. Alongside increases in Lp‐PLA2, E‐selectin was higher and ICAM‐1 tended to be higher in ERBP. Experimental data and clinical observations support a role for cell adhesion molecule (CAM)‐mediated leukocyte adhesion in the development of early vascular lesions (Poston et al. 1992; Price and Loscalzo 1999; Krieglstein and Granger 2001). CAMs are increasingly viewed as critical participants in the vascular dysfunction and tissue injury that are associated with a wide variety of inflammatory and cardiovascular diseases (Krieglstein and Granger 2001; Eikendal et al. 2018). These results suggest that vascular inflammation may be present in ERBP prior to the clinical progression to hypertension, although the mechanisms remain unclear.

Notably, we also observed that E‐selectin and ICAM‐1 levels were positively correlated with MSNA, and E‐selectin levels were negatively correlated with cardiovagal BRS. These findings suggest that vascular inflammation may play a mechanistic role on SNS activation directly, and/or via impaired BRS. Vascular inflammation could modulate baroreflex sensitivity in the brainstem (central integrative or efferent dysregulation) and/or at the level of the baroreceptor nerve endings within the vasculature, thereby perpetuating an increase in arterial BP by maintaining SNS overactivation. Alternatively, E‐selectin and ICAM‐1 may have direct sympathoexcitatory effects, independent of their action on cardiovagal BRS. Taken together, our findings support a link between vascular inflammation and SNS activation, as well as vascular inflammation and blunted cardiovagal BRS in ERBP.

We recognize several limitations to our study. First, the study population was mainly comprised of African‐American men; therefore, the results may not be generalizable to women or other racial groups. Second, we measured systemic biomarkers of inflammation rather than directly from blood vessels; however, Lp‐PLA2, E‐selectin, and ICAM have been shown to reflect vascular inflammation in prior studies (Krieglstein and Granger 2001; Kim et al. 2017). Third, 25% of the variance in increasing MSNA is explained by E‐selectin, and 23% of the variance in increasing MSNA is explained by changes in ICAM‐1. Therefore, factors other than vascular inflammation, such as elevated oxidative stress, activation of the renin‐angiotensin‐aldosterone system, or genetic predisposition could also contribute to sympathetic overactivation in ERBP. Fourth, we did not explore other biomarkers of systemic inflammation, and focused on vascular biomarkers. Fifth, although we report SNS overactivation, dysregulation, and vascular inflammation in ERBP, it is unclear whether these physiological derangements occur prior to the development of ERBP, or if elevated BP leads to the autonomic dysfunction and inflammation. It is also unclear if SNS overactivation and vascular inflammation are associated with accelerated progression to hypertension; this should be evaluated in future longitudinal studies.

In summary, we observed that ERBP in the prehypertensive range is characterized by higher resting MSNA and impaired arterial BRS. Concomitantly, we observed greater levels of the vascular inflammatory biomarkers Lp‐PLA_2_ activity and E‐selectin in ERBP. E‐selectin correlated with impaired cardiovagal BRS in ERBP, suggesting a link between vascular inflammation and impaired BRS in ERBP. Taken together, such autonomic derangements may contribute to increased risk of hypertension and CVD in individuals with ERBP. Future studies should investigate whether therapeutic interventions targeting vascular inflammation and autonomic dysfunction might mitigate the progression to hypertension and development of CVD in ERBP.

## Conflict of Interest

No conflicts of interest, financial or otherwise, are declared by the authors.
